# Fabrication and Characterisation of Stimuli Responsive Piezoelectric PVDF and Hydroxyapatite-Filled PVDF Fibrous Membranes

**DOI:** 10.3390/molecules24101903

**Published:** 2019-05-17

**Authors:** Biranche Tandon, Prashant Kamble, Richard T. Olsson, Jonny J. Blaker, Sarah H. Cartmell

**Affiliations:** 1 School of Materials, MSS Tower, The University of Manchester, Manchester M13 9PL, UK; biranche.tandon@manchester.ac.uk (B.T.); prashant.kamble3654@gmail.com (P.K.); 2 Bio-Active Materials Group, School of Materials, MSS Tower, The University of Manchester, Manchester M13 9PL, UK; 3 Department of Fibre and Polymer Technology, School of Chemical Science and Engineering, KTH Royal Institute of Technology, Teknikringen 56, SE-10044 Stockholm, Sweden; rols@kth.se

**Keywords:** electrospinning, solution blow spinning, piezoelectric, poly(vinylidene fluoride), wettability, surface roughness, tissue repair

## Abstract

Poly(vinylidene fluoride) has attracted interest from the biomaterials community owing to its stimuli responsive piezoelectric property and promising results for application in the field of tissue engineering. Here, solution blow spinning and electrospinning were employed to fabricate PVDF fibres and the variation in resultant fibre properties assessed. The proportion of piezoelectric β-phase in the solution blow spun fibres was higher than electrospun fibres. Fibre production rate was circa three times higher for solution blow spinning compared to electrospinning for the conditions explored. However, the solution blow spinning method resulted in higher fibre variability between fabricated batches. Fibrous membranes are capable of generating different cellular response depending on fibre diameter. For this reason, electrospun fibres with micron and sub-micron diameters were fabricated, along with successful inclusion of hydroxyapatite particles to fabricate stimuli responsive bioactive fibres.

## 1. Introduction

Stimuli responsive polymers are a class of materials capable of responding to one or more stimuli such as mechanical, photo, electrical (physical stimuli), pH, electrochemical gradients (chemical stimuli), enzymes and receptors (biological stimuli) [1,2]. The stimuli responsive nature of these materials has been exploited for applications such as drug delivery and tissue engineering [3]. Poly(vinylidene fluoride) (PVDF) and its copolymers can exhibit piezoelectricity, pyroelectricity and ferroelectricity, and are classified as stimuli responsive polymers. These polymers have been utilised alone or as matrices in composites and layered structures to fabricate stimuli responsive systems [4,5,6]. Tissue engineering scaffolds fabricated using these materials are capable of generating electrical stimuli in response to mechanical deformation, which consequently affect cellular differentiation [7,8,9,10]. The incorporation of particles of hydroxyapatite (HA), itself a piezoelectric material, into polymer and ceramic matrices has been shown to impart bioactive properties, improve bone forming ability and apatite formation [11,12,13,14].

Electrospinning (ES) has been utilised to fabricate nanofibre membranes for various applications such as tissue engineering [15,16], sensors [17,18] and filtration [19] over the past two decades. To date, ES has been the predominant technique used to produce sub-micron fibres from PVDF and its copolymers [8,20,21,22,23]. However, the requirement for high voltage setups, conductive collectors and the relatively low fibre production rates typical of the technique can pose issues for scale-up [24,25]. Solution blow spinning (SBS) has emerged an alternative technique for sub-micron/nanofibre fabrication [24,26], it does not require electric fields and offers high throughput making it attractive for industrial scale-up [27,28]. There have been instances where SBS has been used to fabricate fibrous PVDF [29] and poly(vinylidene fluoride)-*co*-tetrafluoroethylene (PVDF-*co*-TrFE) [30] membranes, albeit very few in comparison to electrospinning. Differences in PVDF-based polymer composition, molecular weight, spinning technique and experimental parameters have been shown to significantly impact the resultant fibres [20,31,32,33,34].

Difference in fibre diameter influences the roughness and inter-fibre pore sizes of membranes and scaffolds used in tissue engineering applications and can have a direct influence on cellular adhesion, proliferation and differentiation [35,36,37]. Controlling fibre size is a strategy that can be used to tune pore size and mimic aspects of the extracellular matrix (ECM) to alter cell infiltration [38]. This approach has been shown to enable the migration of human osteosarcoma cells (SaOs-2 cell line) from one side of a fibre membrane to the other, and support their proliferation [38]. The differentiation and spreading of osteoblastic cell line, MC3T3-E1 cells has also been reported to be affected by fibre diameter [37]. 

In this work, the effect of PVDF molecular weight and spinning technique on fibre properties were investigated, specifically, fibre morphology, diameter distribution, mat quality, production rate, thermal, wettability, mechanical and piezoelectric properties, as well as the effect of HA particulate inclusion within the fibres. The properties of the PVDF fibre mats were also compared to films of non-polarised (non-piezoelectric) and polarised (piezoelectric) PVDF. Batch-to-batch variation analysis was also conducted to assess the reproducibility between the two spinning techniques. 

## 2. Results and Discussions

**Figure 1:**
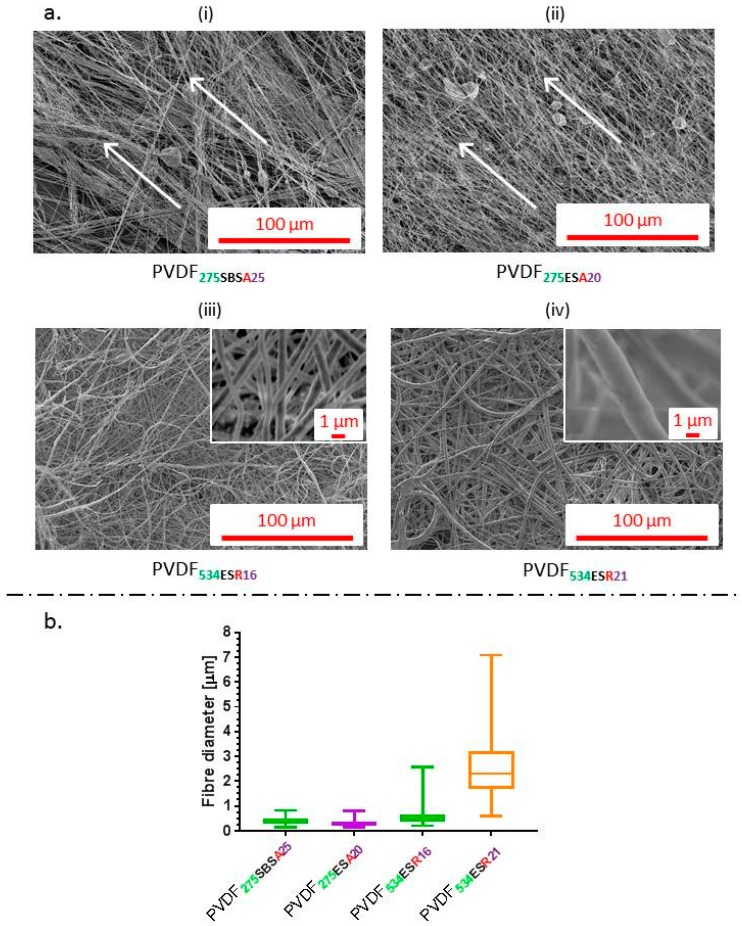
(**a**) Scanning electron microscopy (SEM) images of aligned and random fibres obtained using SBS and ES. White arrows represent the direction of alignment. (**b**) Fibre diameter distribution of the different fibrous membranes fabricated using SBS and ES.

Representative scanning electron microscopy (SEM) images of the fibre mats are shown in Figure 1a. Fibre alignment, in the direction of the rotating collector was evident (Figure 1a (i) and (ii)). Alignment of ES fibres was apparently higher than those produced by SBS in this study (Figure 1a). This could be due to increased turbulence around the collector due to high rotation speed [39] and compressed air deflecting from the surface of the cylindrical collector. Mean fibre diameters of 400 ± 130 nm and 300 ± 90 nm were obtained for SBS and ES, respectively. All fibres exhibited relatively smooth morphologies. The fibre diameter and morphology observed for PVDF fibres was similar to previously reported for ES PVDF [40,41]. Previous studies concerning fibre spinning of PVDF using ES and SBS yielded similar results supporting successful fabrication of nanofibrous PVDF membranes [20,22,23,29]. The presence of beads in PVDF**_275SBSA25_** was in accordance with previous results obtained with SBS of PVDF-*co*-TrFE [30] and may stem from a local higher concentration of polymer and charge accumulation [42].

**Figure 2:**
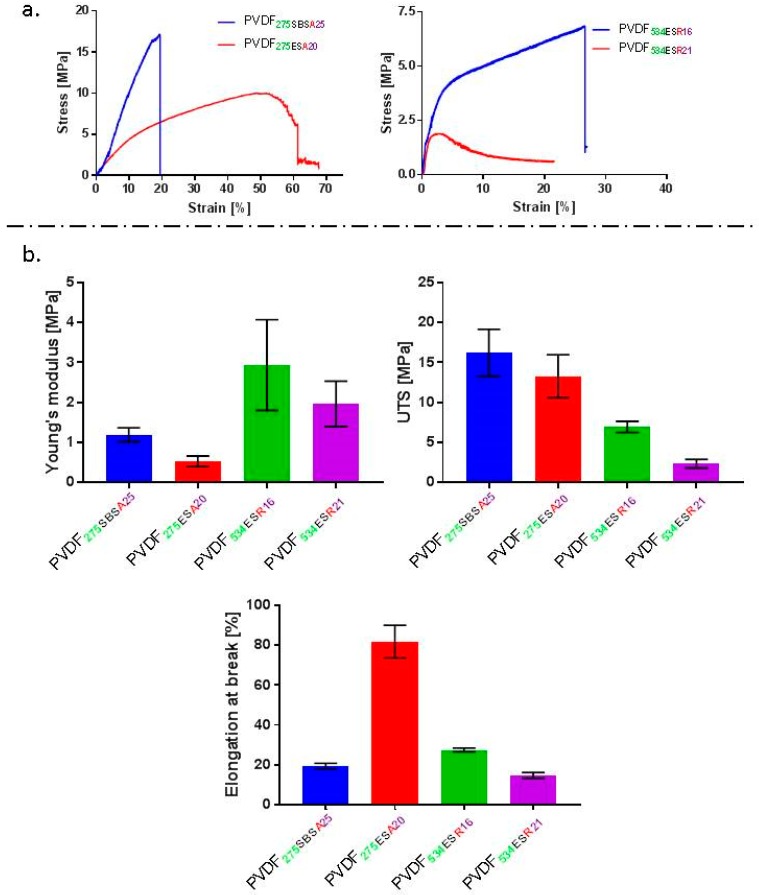
(**a**) Representative stress-strain curves for different fibres fabricated using SBS and ES, (**b**) graphs presenting values calculated for different mechanical properties such as Young’s modulus, ultimate tensile strength (UTS) and elongation at break for fabricated fibres.

Tensile testing revealed PVDF**_275SBSA25_** fibre mats to have higher tensile strength (16.2 MPa) and Young’s modulus (1.2 MPa) in comparison to PVDF**_275ESA20_** fibre mats (13.3 MPa and 0.5 MPa respectively) (Figure 2b). Fibrous membranes obtained in this study had higher tensile strength and lower Young’s modulus than those previously reported for ES PVDF fibres [33]. Direct comparison is however difficult as fibre mat mechanical properties can be affected by the composition of solvent and the molecular weight of PVDF being utilised to spin fibres [33,43,44]. Furth to this, inter fibre pore size, mat/membrane porosity and fibre diameter influence mechanical properties [45,46]. The elongation at break% was found to be higher for PVDF**_275ESA20_** fibres, i.e., circa 80% strain at break compared to 20% for the solution blow spun fibres. A contrary trend in failure behaviour was observed by Bolbasov et al. in their study of mechanical properties of nonwoven SBS and ES membranes of PVDF-*co*-TrFE [47]. The uniaxial tensile strength of non-woven fabrics in their study was higher for ES scaffolds [47]. This discrepancy could arise from a difference in alignment of fibres and the composition of the polymer used. PVDF is known to be a chemically resistant and hydrophobic polymer, the mechanical properties of which are not susceptible to changes in hydration, provided that any solvents used in processing have been sufficiently removed [32,48]. The results from tensile testing are summarised in Figure 2.

**Figure 3:**
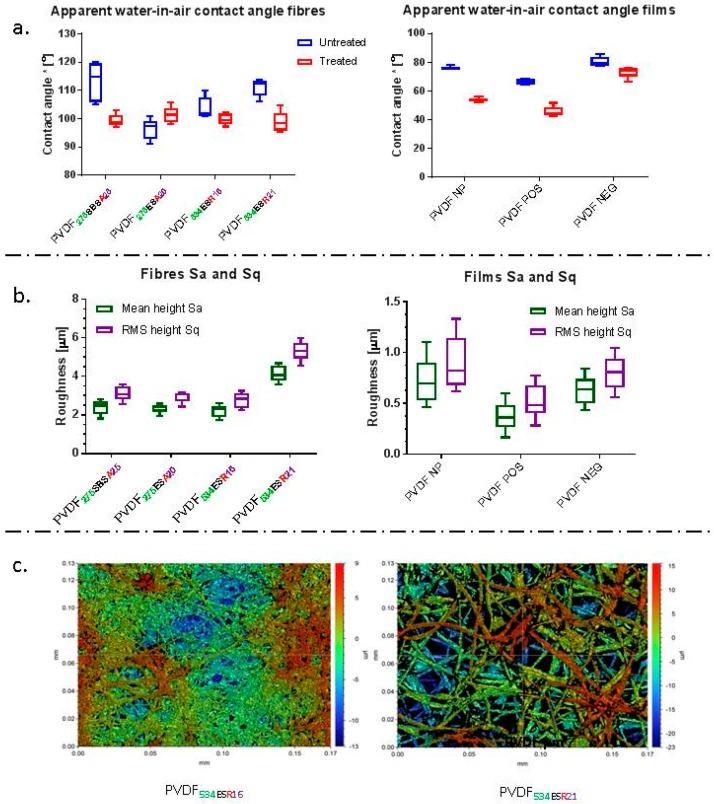
(**a**) Apparent water-in-air contact angle values obtained for untreated and treated (pre-conditioned with cell culture media and dried) fibre mats and films, * represents apparent water-in-air contact angle (**b**) Distribution of roughness parameters obtained for fibrous PVDF membranes and PVDF films. (**c**) Representative optical profilometry images obtained for different samples.

Apparent water-in-air contact angle measurements showed no significant difference in wettability between the pre-conditioned scaffolds (treated overnight with cell culture media) and untreated SBS fibre membranes. All fibrous membranes exhibited hydrophobic character with contact angle values >90° [41,49]; untreated SBS fibre membranes had a higher average contact angle of ~113°, in comparison to the trend observed for ES membranes and all treated membranes (Figure 3a). The contact angle values of ~100° observed for treated samples were lower than that of the non-treated fibre groups (Figure 3a.). Preconditioning of the samples can lead to nonspecific protein adsorption depending on initial wettability and consequently affect the response of cells seeded on the scaffolds [50]. PVDF**_275SBSA25_** fibre mats were observed to be hydrophobic (in the absence of pre-conditioning) with a mean contact angle of 113°, which reduced to 99° after preconditioning with cell culture media and drying. This reduction was not observed for the electrospun PVDF**_275ESA20_** fibre mats, which presented a contact angle of ~100°. The hydrophobic character of PVDF fibres is well supported by different studies aimed at the fabrication of filtration membranes [51,52,53]. However, although the membranes were found to be initially hydrophobic, for preconditioned samples the contact angle values reduced to < 90° after 30 s. Pre-conditioning of fibre membranes of different polymers has previously been shown to reduce the contact angle and improve the wettability of scaffolds prior to cell culture [54,55]. The roughness of fibre membranes and the fibres themselves can also influence wettability [56]. Contact angle values can also be influenced by the composition of solvents used during spinning and the type of polymer being spun [57,58]. Techniques such as low-pressure, low-temperature plasma treatment can be applied for altering surface chemistry, topology and hence wettability of fibre membranes [59,60,61].

Interferometric optical profilometry was used to assess the surface topography and obtain surface roughness. The overall surface roughness of the SBS fibre mats (S_a_ = 2.4 μm and S_q_ = 3.1 μm) and ES fibre mats (S_a_ = 2.3 μm and S_q_ = 2.8 μm) was higher than those observed for films (Figure 3b). The fibre membranes present a hydrophobic character and higher roughness than smooth/film type surfaces for PVDF, which correlates well with the hydrophilic character of films as depicted by contact angle values [56,62,63]. Fibre collection method, electrical field strength applied during spinning, and fibre alignment, are also known to influence substrate roughness and wettability [64]. The surface roughness values recorded for the PVDF fibre mats in this work were significantly higher than those observed for smooth control films. PVDF NP (non-poled PVDF films), PVDF POS (positive surface of poled/piezoelectric PVDF film) and PVDF NEG (negative surface of the poled/piezoelectric PVDF film) had S_a_ (S_q_) values of 0.7 (0.9) μm, 0.4 (0.5) μm and 0.6 (0.8) μm respectively. 

**Figure 4:**
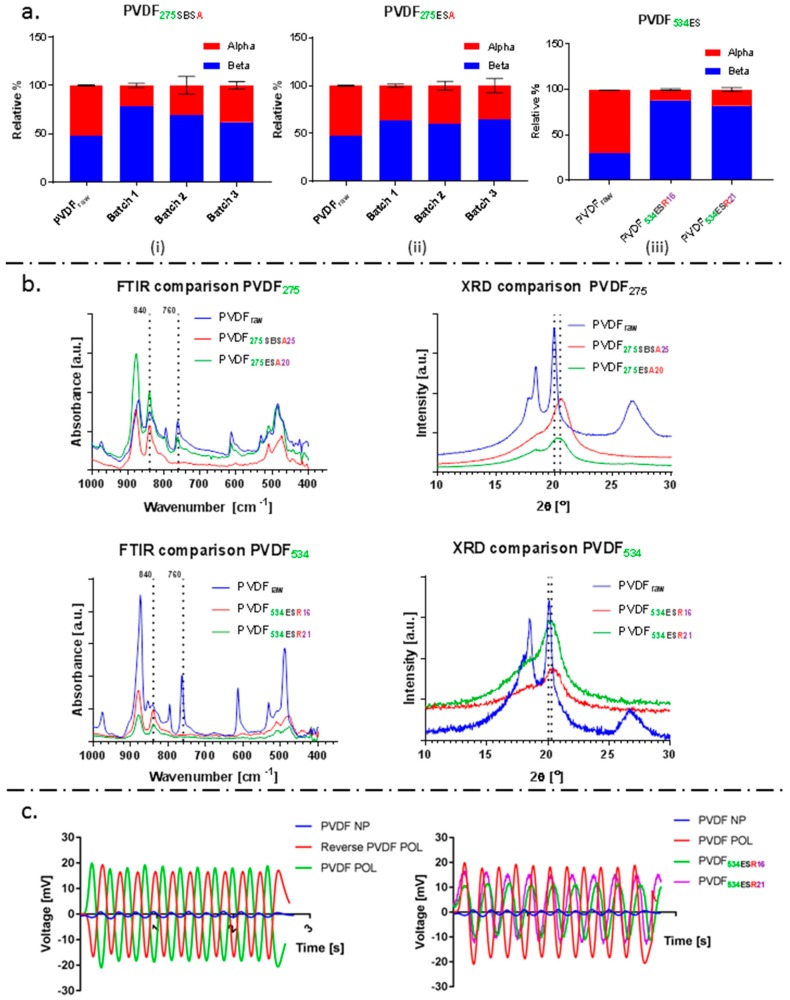
(**a**) Batch-to-batch variation of the relative% of α- and β-phase in (i) SBS fibre mats, (ii) ES fibre mats, and (iii) amount of α- and β-phase present in micron and sub-micron ES fibre mats in comparison to raw PVDF. (**b**) Representative FTIR and X-RD graphs of the different fibre mats fabricated. Vertical dotted lines in FTIR graphs represent the characteristic peaks for α- (760 cm^-1^) and β- (840 cm^-1^) phase. A peak shift from ~2*θ* = 20° to ~2*θ* = 20.3° and the suppression of peaks at ~2*θ* = 18.5° and ~2*θ* = 26.7° determined for the fibre mats is evidence of conversion from α-phase to the electroactive β-phase when the raw polymer is processed into fibres. (**c**) Piezoelectric voltage response obtained for control PVDF films and comparison to the response obtained for the fibre mats fabricated using PVDF**_534_**. A peak-to-peak voltage of 2.25 mV, 33.64 mV, 21.94 mV and 27.24 mV was observed for PVDF NP film, PVDF POL film, PVDF**_534ESR16_** and PVDF**_534ESR21_** fibre mats, all respectively.

Following physical and morphological characterisation of the materials, the emphasis was shifted to test the reproducibility of the fibre membranes and their piezoelectric characteristics. FTIR revealed the characteristic peaks of β-phase (840 cm^-1^) and no prominent peaks for γ-phase (833 cm^−1^ and 1233 cm^−1^) for both types of fibres fabricated [65,66], as shown in Figure 4b. X-RD results further confirm the presence of β-phase in the fibre membranes. Quantification of the electroactive β-phase yielded a higher value for SBS fibre mats when compared to those of electrospun fibres, and found similar to that of commercially obtained PVDF piezoelectric films (PVDF POL) (SBS ~76% (PVDF**_275SBSA25_**); ES ~67% (PVDF**_275ESA20_**), and ~75% for PVDF POL). FTIR evaluations obtained are summarised in Figure 4. The higher solution flow rate of 75 μL min^−1^ and 25% *w*/*v* concentration used in SBS results in > 300% higher throughput of PVDF fibres as compared to ES (as summarised in Table 1). However, batch-to-batch comparison of the β-phase content revealed lower variability in the fibres fabricated using ES. SBS fibre mats exhibited a variation in the amount of β-phase of ~15%, compared to ES fibre mats at ~5%. Solvent evaporation rate and environmental temperature during processing are known to influence the content of piezoelectric phase in PVDF-based materials [67,68] and could account for the greater variability observed in SBS fibre mats. PVDF solution temperature, air temperature, pressure, flow, and spinning environment, in addition to polymer molecular weight, influence spinnability [69]. This highlights the importance of controlling the temperature of PVDF solution and spinning environment used to fabricate PVDF fibres and warrants further investigation. The percentage crystallinity of fibres and films was in the range ~40–45%, as determined by differential scanning calorimetry (DSC), summarised in Table 2. 

**Figure 5:**
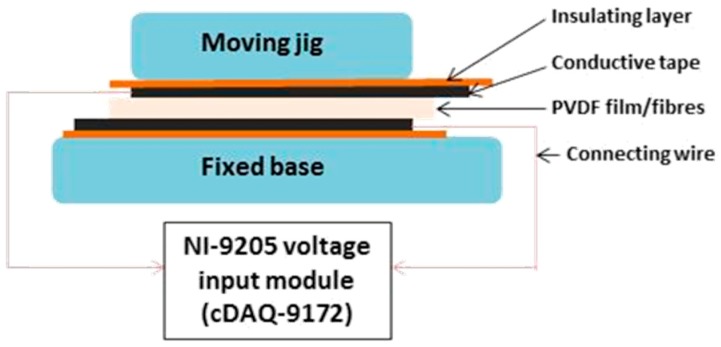
Schematic of the piezoelectric voltage measurement setup.

Increasing the concentration of PVDF**_275_** did not have a considerable effect on fibre diameter using electrospinning. It was possible to produce sub-micron fibres, yet not micron-size fibres, therefore PVDF**_534_** was used as increasing molecular weight has been shown to increase fibre diameter [31]. Fibres with mean fibre diameter of 550 ± 300 nm (PVDF**_534ESR16_**) and 2.5 ± 1.1 μm (PVDF**_534ESR21_**) were fabricated (Figure 1). The β-phase composition of the fibre mats obtained was higher for PVDF**_534_** in comparison to those of PVDF**_275_** hinting at the possible effect of molecular weight and the field strength conditions of electrospinning on β-phase formation. Hu et al. showed that β-phase content varied with the applied voltage during electrospinning and the value peaked at around 20 kV [21]. In another study, the dependence of β-phase content on the molecular weight of the polymer has been discussed [66]. To test the stimuli responsive characteristic, the piezoelectric characteristics of films and fibres were confirmed by measuring the amount of voltage generation for a given cyclic force (5N) on respective membranes in sandwich configuration (schematic as shown in Figure 5). A reversal in voltage (as shown in Figure 4c) was observed when the electrodes were swapped; this approach ensured that the signal measured was generated as a result of piezoelectricity. 

Results from optical interferometry reveal that the roughness of fibre mats consisting of micron fibres was significantly higher than those of fibre mats formed of sub-micron fibres, and PVDF films (Figure 3). Milleret et al. observed a similar trend of average roughness for sub-micron and micron fibre-based membranes [70]. The higher average surface roughness of micron-sized fibre mats compared to that of other fibre membranes had no effect on the contact angle of scaffolds in the present work. A similar trend of increase in roughness has previously been reported for PVDF fibres [71]. Whilst the mats produced in the current study were more suitable as membranes, such as those for cell guidance [72,73]. Electrospun membranes are usually composed of densely packed fibres with limited inter-fibre spacing and pore size [74,75], typically the through thickness porosity is insufficient to allow for significant cell migration [76,77]. Some techniques for improving the through thickness macroporosity in electrospun membranes have been developed, as reviewed [76,77]. Some of these techniques include cryogenic spinning of fibres [78], laser drilling [79], , as well as the use of sacrificial fibres [75] and in situ porosifiers to achieve interconnected macropores throughout the scaffold to improve cellular infiltration and enhance vascularization [74]. The pore size and inter-fibre spacing of such fibrous membranes can be tailored to the desired application; however, densely packed membranes also have a role as cell guidance substrates and in forming barriers in applications such as wound dressing and preventing infection (e.g., dental applications) [72,73]. 

**Figure 6:**
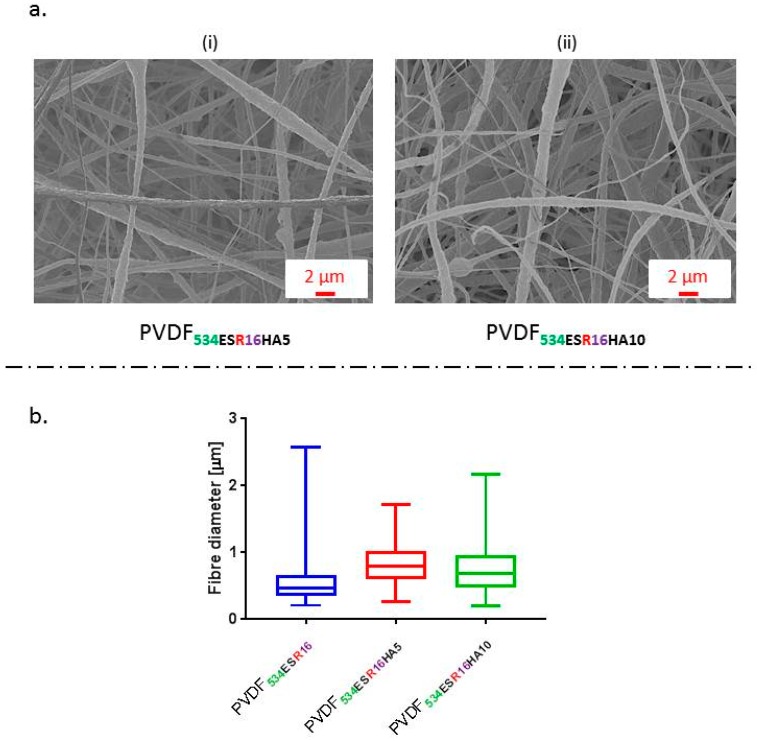
(**a**) SEM images of composite fibre mats obtained by inclusion 5 wt.% (i) and 10 wt.% (ii) HA in PVDF, (**b**) fibre diameter distribution of the ES composite fibres in comparison to neat PVDF fibres.

Hydroxyapatite was incorporated as a filler in the fibres as it is known to enhance the bioactivity of the stimuli responsive scaffolds [14,80]. SEM images of the HA/PVDF composite sub-micron fibres obtained are shown in Figure 6a. Fibres filled with HA exhibited an increase in mean fibre diameter to ~700 nm, compared to neat PVDF ~550 nm. A similar trend has been reported for HA/poly(lactide-*co*-glycolide) composite fibres [81]. The diameter increase could be attributed to changes in properties of the spinning solution, in particular increased viscosity with particle loadings, as well as conductivity, and changes in solvent evaporation rate [82]. X-RD diffraction spectra (Figure 6a) of composite fibres confirmed the incorporation of HA particles. The increase in peak intensities at 2*θ* of 25.9°, 32.2° and 32.9° correspond to (002), (112) and (300) (respectively), typical of HA (International Centre for Diffraction Data (ICDD) 9-432), in PVDF**_534ESR16HA10_** samples when compared with PVDF**_534ESR16HA5_** samples is indicative of higher filler inclusion [82]. 

**Figure 7:**
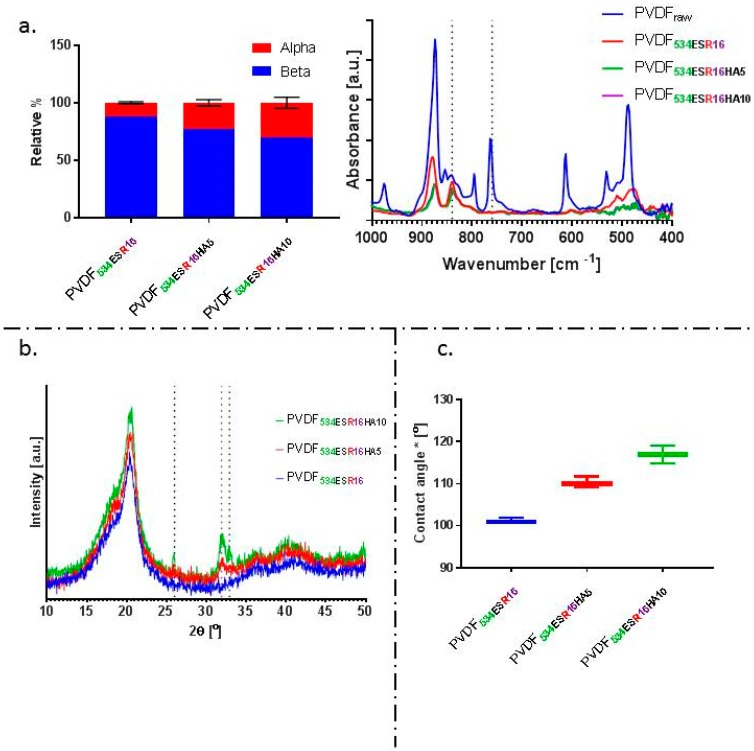
(**a**) Relative% of α- and β-phase and representative FTIR spectra of electrospun HA/PVDF composite fibre mats. A reduction of β-phase from 88.02% in PVDF**_534ESR16_** fibres to 77% and 70% was observed for PVDF**_534ESR16HA5_** and PVDF**_534ESR16HA10_** composites, respectively. (**b**) X-RD graphs of the composite fibres obtained. (**c**) The apparent water-in-air* contact angle of the composite fibre mats obtained.

Inclusion of HA led to a significant reduction in net β-phase content of the composite nanofibre membranes (determined by FTIR analysis). Average β-phase content of 77% and 70% was observed for PVDF**_534ESR16HA5_** and PVDF**_534ESR16HA10_**, respectively. Further, percentage crystallinity data obtained from DSC (shown in Table 2) suggests that the overall crystallinity of the composite fibres was significantly reduced compared to PVDF fibres. The apparent water-in-air contact angle increased with the amount of HA in fibres (Figure 7c.). PVDF**_534ESR16HA5_** and PVDF**_534ESR16HA10_** had contact angle values of ~110° and 117°, respectively, and presented more hydrophobic character than PVDF**_534ESR16_** (~100°). A similar trend was reported by Brage et al. for HA filled PVDF composites [83]. A reduction in crystallinity and β-phase in composite fibres reduces the net amount of crystalline electroactive phase suggesting reduced piezoelectric character of the polymer. This effect could be offset by the improvement in bioactivity due to the presence of HA, itself a piezoelectric material [84].

## 3. Experimental

### 3.1. Materials

PVDF of weight average molecular weight (Mw) 275 kg mol^−1^ (medium molecular weight, denoted throughout as PVDF**_275_**) and 534 kg mol^−1^ (high molecular weight, denoted throughout as PVDF**_534_**) were purchased from Sigma Aldrich (Gillingham, UK) and supplied in pellet and powder form, respectively. Organic solvents used for spinning, *N, N*-dimethyl formamide (DMF) and acetone were purchased from VWR international limited, and Fisher Scientific (Loughborough, UK) respectively. Hydroxyapatite (HA, particle size distribution shown in Figure S1) was obtained from Sigma Aldrich, UK. More than 75% of the HA particles were found to be < 400 nm in size 

Films of piezoelectric PVDF (poled, 28 μm thickness) and non-piezoelectric (non-poled, 16 μm thickness) were purchased from Precision Acoustics Ltd., (Dorchester, UK). The piezoelectric strain constants of the PVDF piezoelectric films were 22 pC/N (d_31_), 3 pC/N (d_32_) and −30 pC/N (d_33_) according to the manufacturer’s data sheet.

### 3.2. PVDF and Composite Hydroxyapatite-Filled PVDF Fibre Spinning

PVDF was dissolved either in DMF or a mixed solvent of DMF and acetone (1:1 solvent volume ratio) at various concentrations (between 16 and 25% *w*/*v*), as given in Table 1. This mixed solvent approach has been used to fabricate PVDF fibres via electrospinning [20]. The presence of acetone with DMF reduced the prevalence of beads and facilitates solvent evaporation during fibre spinning [20]. PVDF/DMF and PVDF/mixed solvent solutions were stirred at 70 °C and 50 °C, respectively, in capped vials, using a magnetic hotplate stirrer (~300 rpm). The lower temperature (50 °C) was used for the mixed solvent system to mitigate unwanted, premature evaporation of acetone from the solution. The polymer was observed to completely dissolve in ~4 h and was then transferred to a 5 mL syringe and used for electrospinning or solution blow spinning.

Solution blow spinning (SBS) was conducted using equipment previously described [78], here an air pressure of 35 psi was applied and a PVDF concentration of 25% *w*/*v* was spun into fibres. A blunt stainless steel 18G needle was used as an inner nozzle through which the solution was injected. The protrusion of the inner nozzle beyond the outer nozzle was 8–10 mm. The choice for the solvent system (DMF only) and polymer concentration (25% *w*/*v*) was made following optimisation of various DMF: acetone ratios (for solvent) and concentrations of polymer (not reported here). 

Electrospinning (ES) was conducted on both medium and high molecular weight PVDF using the mixed solvent between 16% and 21% *w*/*v* (polymer mass to solvent volume) in an effort to produce sub-micron and micron-sized PVDF fibres. The ES equipment used was similar to the one previously described [49]; polymer solutions were injected through a blunt stainless steel 21G needle with the positive electrode attached. Concentrations above 21% *w*/*v* lead to interrupted spinning and gelification of the precursor solution at the needle tip and were not investigated. 

Fibres were collected using various fibre-emitter to collector distances (working distance of between 15 cm to 17.5 cm for ES, and 30 cm for SBS) and various rotating collector speeds in an effort to attain random and aligned fibre mats. Control over the alignment of fibres was established by using a high-speed rotating disk for fibre collection [20]. Hydroxyapatite (HA)-filled PVDF composite fibres were fabricated by ES using PVDF**_534_** at 16% *w*/*v* polymer concentration. HA particles were loaded at 5% and 10% (wt.% = (mass HA)/(mass HA + mass polymer) × 100), mixed into the solvent and ultrasonicated prior to addition of the polymer to give a 16% polymer mass to solution volume. Ultrasonication was performed using a Branson Digital Sonifier (probe sonication) for 8 min with on-off pulse durations of 5 s. Solutions were prevented from heating due to sonication by placing the vials in an ethanol bath. Processing parameters are detailed in Table 1.

### 3.3. Characterisation of the Fibre Mats

#### 3.3.1. Fibre Morphology, Fibre Diameter and Size Distribution

Fibre morphology was assessed via scanning electron microscopy (SEM; Hitachi high technologies S-3000N, Tokyo, Japan). Fibre mat sections were cut, mounted and sputter coated as previously described [82]. Fibre diameters were determined using ImageJ, (version 1.50i, NIH, Bethesda, Maryland, USA); at least 100 individual fibre diameters were measured per sample type.

#### 3.3.2. Mechanical Properties of the Fibre Mats

Uniaxial tensile tests were conducted on fibre mats using an Instron 3344 Universal Testing System. Fibres from PVDF**_275_** (aligned) were directly collected on small card frames (windows 30 mm length × 10 mm width, and frame edge 2.5 mm width) while fibre mats from PVDF**_534_** (random) were affixed to bigger frames (windows 45 mm × 20 mm width, and frame edge 5 mm width). The smaller frames were utilised for aligned fibre mats due to difficulties in handling. The ends of fibre mats were taped on to the frames using double-sided tape and a card tab was taped over the double-sided tape to provide better grip and avoid failure due to stress from the clamps [85]. The effective dimensions of tested fibre mats were 20 mm length × 5 mm width (small frames) and 25 mm length × 10 mm width (large frames). Frame edges were cut prior to mechanical testing of the fibre mats. All samples (*n* = 6 per sample type) were tested at 2 mm min^−1^ using a 10 N load cell [85]. Force-extension data was recorded during measurement and stress was calculated using equation (1) given below, to assess the effective solid content. The ultimate tensile strength (UTS), Young’s modulus and percentage elongation at break were recorded for each fibre mat type.

(1) $$Stress=\frac{Force}{A*s}$$

where *A* is the area of the cross-section of the fibre mat and *s* is the effective solid polymer content calculated as (1 − porosity).

Porosity measurements were conducted on the fibre mats in order to determine stress (due to solid content) and piezoelectric voltage per unit material (described further below). Fibre mats were cut into rectangular samples with a specific size (see above). These mats were weighed and their average thickness determined by measuring with a digital micrometre (in three places). Porosity was determined using Equation (2) below ([86]):

(2) $$Porosity\;\%=\left(1\;-\left(\frac{M_{s}}{\rho V_{s}}\right)\right)\;\times \;100$$

where *M_s_* is the mass of fibre membrane of a given size, *ρ* is the density of PVDF and *V_s_* is the volume (fibres + void spaces) of the sample calculated by multiplying length, width and average thickness (thickness measured at three different points on the mat) of the fibre mats before mounting them on window frames for mechanical testing.

#### 3.3.3. Wettability of the Fibre Mats by Water-in-Air Contact Angle Analysis

Static contact angle measurements were conducted using a Krüss Drop Shape Analyzer (DSA 100). At least 10 different measurements were performed per sample type, with and without exposure to cell culture medium and drying (treated fibres). Contact angle values were recorded over time (up to 1 min) and the values observed at t < 5 s were utilised for all sample types. Cell culture media were prepared by supplementing minimum essential media (MEM-α, Sigma Aldrich, UK) with 10% foetal bovine serum and 1% each of L-glutamine and antibiotic-antimycotic solution. PVDF films and fibre mats were affixed to glass coverslips (12 × 12 mm) using adhesive (commercial silicone sealant, Dow Corning 732) to ensure that the substrates were flat; care was taken to ensure that the adhesive did not influence the contact angle (e.g., membranes of sufficient thickness). Pre-conditioned substrates (treated) were soaked in ethanol for one hour. Ethanol was removed and the treatment was followed by overnight conditioning in cell culture media. This pre-treatment approach was followed in order to imitate the procedures used in preparation for cell culture. Substrates used without the above treatment are referred to as untreated substrates. 

#### 3.3.4. Surface Roughness Determination by Optical Profilometry

Optical profilometry was used to determine the roughness of the fibre mats, known to influence the initial adhesion and spreading of cells [38,87]. A Contour GT-K1 white light interferometer (Bruker, Massachusetts, United States) was used in vertical scanning interferometry (VSI) mode. Samples were prepared as described above in Section 3.3.3. Mean surface mean height (S_a_) and root mean square height (S_q_) were calculated using Vision64 analytical software. A total of 10 images were taken per sample type (analysed area for each image: 170 μm × 130 μm for fibres and 90 μm × 65 μm for films), analysis was conducted on each image, and average values for mean surface mean height (S_a_) and root mean square height determined for each sample type.

#### 3.3.5. Determination of Electroactive Phase by ATR-FTIR Analysis 

Attenuated total reflectance Fourier transform infrared spectroscopy (ATR-FTIR, FTIR Nicolet iS5; Thermo Scientific, UK) was performed to quantify the amount of electroactive phase present in the samples. Each measurement reported is based on 32 scans at 4 cm^−1^ in the range of wavenumber 400–1500 cm^−1^. The characteristic peaks for β-phase were at wavenumbers 510, 840 and 1279 cm^−1^ and those for α- phase were at 408, 532, 614, 766, 795, 855 and 976 cm^−1^ [65,88]. A method reported in Reference [88] was used to quantify the relative amount of electroactive phase present in the sample, using Equation (3) below.

(3) $$F_{EA}=\frac{A_{840}}{\left(\frac{K_{840}}{K_{766}}\right)A_{766}+A_{840}}\;\times \;100$$

where *A*_766_ and *A*_840_ are the absorbance values at 766 and 840 cm^−1^, respectively. The values of *K*_766_ and *K*_840_ (absorption coefficients at respective wavenumbers) are 6.1 × 104 and 7.7 × 104 cm^2^ mol^−1^, respectively [88]. 

#### 3.3.6. X-ray Diffraction Analysis

X-ray diffraction (X-RD) was used to qualitatively validate and assess the formation of electroactive β-phase. X-RD diffraction spectra were obtained using a PANalytical X’Pert PRO X-ray diffractometer with Cu-K_α_ radiation (wavelength 0.154 nm) operated at 40 kV and 40 mA. Both fibre and film samples were scanned in the 2*θ* range of 4° to 50° with a step interval of 0.03342°. 

#### 3.3.7. Qualitative Determination of Piezoelectric Characteristics

A rig was constructed to record voltage generated from the piezoelectric materials on mechanical deformation, schemed in Figure 5. A cyclic loading regime of 5 N with a frequency of 4 Hz was applied using an Instron 3344 mechanical testing instrument; similar to approaches previously reported to test the piezoelectric characteristics of PVDF fibre mats [21,34]. Contact surfaces were insulated using electrical insulating tape to ensure that the electrical signals generated were collected and measured efficiently. A voltage input module (NI 9205, National Instruments Corporation (U.K.) Ltd.) was used following manufacturer instructions. Porosity measurements (described above) were utilised to obtain the amount of voltage generated by the effective amount of PVDF present (normalised voltage) in the fibre mats. The normalised voltage values were compared to those obtained for PVDF films. Data was collected using LabVIEW software and cyclic voltage response was re-plotted using GraphPad Prism (Version 7.04).

#### 3.3.8. Thermal Characterisation

Differential scanning calorimetry (DSC) measurements were performed on films, fibres and raw PVDF to determine the effect of processing on melt temperature (T_m_) and crystallinity. A heat–cool–heat regime was applied, from room temperature to 200 °C at 10 °C min^−1^, then cooled to −50 °C at the same rate, and reheated as aforementioned. The degree of crystallinity (Δ*X_c_*) of each sample is directly proportional to the value of enthalpies (Δ*H_m_*) obtained [89]. Equation (4) was used to calculate the percentage crystallinity of the samples.

(4) $$\Delta X_{C}=\;\frac{\Delta H_{m}}{x\;\Delta H_{\alpha }+y\;\Delta H_{\beta }}$$

where Δ*H_m_* is the melting enthalpy of the sample, Δ*H_α_* (93.07 J g^−1^) and Δ*H_β_* (103.4 J g^−1^) are the melting enthalpies for samples in 100% α- and β- phase, respectively [89]; *x* and *y* indicate the fractional content of respective phases in the sample.

The melting enthalpy for composite HA/PVDF fibres was calculated using Equation (5). The melting enthalpy was corrected for the amount of PVDF present in composite scaffolds by dividing it by the weight fraction (*w*) of the polymer present [90,91].

(5) $$\Delta X_{C}=\;\frac{\left(\left(\Delta H_{m}\right)/w\right)}{x\;\Delta H_{\alpha }+y\;\Delta H_{\beta }}$$

## 4. Conclusion

In this work, solution blow spinning and electrospinning have been used to fabricate PVDF fibrous membranes, consisting of different target fibre diameters. The piezoelectric β-phase of fibres has been investigated and compared to that of PVDF films. A comparison across batches of fibres fabricated has been performed to test the reproducibility of the fibres. Fibre mat physical properties were also characterised and important aspects such as roughness and wettability were assessed with respect to future application in cell culture. The piezoelectric characteristics of fibre membranes have qualitatively been demonstrated by measuring the voltage output obtained in response to mechanical deformation. 

Comparison of the characteristics of stimuli responsive PVDF fibres showed that solution blow spinning enabled higher fibre production rates of >300% than electrospinning and also higher β-phase content in the fabricated fibres. Electrospinning was shown to have lower variation in the amount of β-phase in a batch-to-batch analysis and enabled a wider range of fibre diameters (mean diameter of ~550 nm to ~2.5 μm) to be targeted and spun. Stimuli responsive piezoelectric characteristics alongside quantification of crystallinity and electroactive phase elucidated the effect of spinning technique and parameters on these properties. Successful inclusion of the known piezoelectric ceramic and bioactive phase, hydroxyapatite, at 5 wt.% and 10 wt.% within the fibres was achieved. This study provides insight into the important considerations to be made in spinning PVDF fibres and bioactive phase loaded PVDF and their properties, towards application in tissue engineering.

## Figures and Tables

**Table 1:** Summary of polymer solutions and fibre spinning parameters investigated. Note that poly(vinylidene fluoride) PVDF**_534ESR16_** is indicative of PVDF Mw 534 kg mol^−1^ (in green) spun using electrospinning (spinning technique in black) into random (R in red, as opposed to aligned, A in red) using a 16% *w*/*v* solution (in purple).

Spinning Method and PVDF Solution Composition	Solution Flow Rate (μL min^−1^)	Theoretical (dry) Fibre Production Rate (g h^−1^)	Working Distance (cm)	Collection Speed	Voltage Applied (kV)
Solution blow spun PVDF_**275SBSA25**_ in DMF at 25% *w*/*v*	75	1.125	30	1000 rpm (diameter 5 cm); equivalent linear speed 5.236 m s^−1^, aligned fibres	None
Electrospun PVDF_**275ESA20**_ in DMF: acetone (1:1 solvent volume ratio) at 20% *w*/*v*	25	0.3	15	500 rpm (diameter 10 cm); equivalent linear speed 5.236 m s^−1^, aligned fibres	15
Electrospun PVDF_**534ESR16**_ in DMF:acetone (1:1 solvent ratio) at 16% w/v	25	0.24	17.5	50 rpm (diameter 10 cm); equivalent linear speed 0.5236 m s^−1^, random fibres	17.5
Electrospun PVDF_**534ESR21**_ in DMF:acetone (1:1 solvent ratio) at 21% *w*/*v*	25	0.315	17.5	50 rpm (diameter 10 cm); equivalent linear speed 0.5236 m s^−1^, random fibres	7.5
Electrospun PVDF_**534ESR16HA5/10**_ in DMF:acetone (1:1 solvent ratio) at 16% *w*/*v* containing HA at 5 wt.% and 10% wt.%	25	0.24	17.5	50 rpm (diameter 10 cm); equivalent linear speed 0.5236 m s^−1^, random fibres	17.5

**Table 2:** Thermal properties of the fibrous membranes and respective raw materials assessed via DSC. T_m_ and T_c_ are melting temperature (for 1st and 2nd heating profiles) and the temperature of cold crystallisation, respectively.

Material	Tm (°C) 1st	Tm (°C) 2nd	Crystallinity%	Tc (°C)
PVDF pellet	175.2	173.6	45.8	137.3
PVDF powder	159.1	159.7	37.5	127.8
PVDF_**275SBSA25**_	168.6	172.0	45.3	141.6
PVDF_**275ESA20**_	168.3	172.3	42.9	142.3
PVDF_**534ESR16**_	158.5	163.7	42.7	130.0
PVDF_**534ESR21**_	157.4	163.8	45.2	129.6
PVDF_**534ESR16HA5**_	158.9	163.8	36.6	135.2
PVDF_**534ESR16HA10**_	158.9	162.1	38.5	134.8
PVDF NP	175.5	175.4	44.1	151.5
PVDF POL	168.1	172.0	45.6	149.9

## Supplementary Materials

The Supplementary Materials are available online.
