# Supplementary material for: Fabrication and Characterisation of Stimuli Responsive Piezoelectric PVDF and Hydroxyapatite-Filled PVDF Fibrous Membranes
Source: Molecules. 2019 May 17;24(10):1903. doi: 10.3390/molecules24101903 (PMC6571942; doi:10.3390/molecules24101903)
Supplement: Supplementary file 1 [file molecules-24-01903-s001.pdf]

Supplementary Information

Fabrication and characterisation of stimuli responsive piezoelectric PVDF and hydroxyapatite-filled PVDF fibrous membranes

Biranche Tandon [1, 2], Prashant Kamble [1], Richard T. Olsson [3], Jonny J. Blaker [1,2*], Sarah H. Cartmell [1*]

[1] School of Materials, MSS Tower, the University of Manchester, Manchester M13 9PL, UK

[2] Bio-Active Materials Group, School of Materials, MSS Tower, the University of Manchester, Manchester M13 9PL, UK

[3] Department of Fibre and Polymer Technology, School of Chemical Science and Engineering, KTH Royal Institute of Technology, Teknikringen 56, SE-10044 Stockholm, Sweden

* Corresponding authors

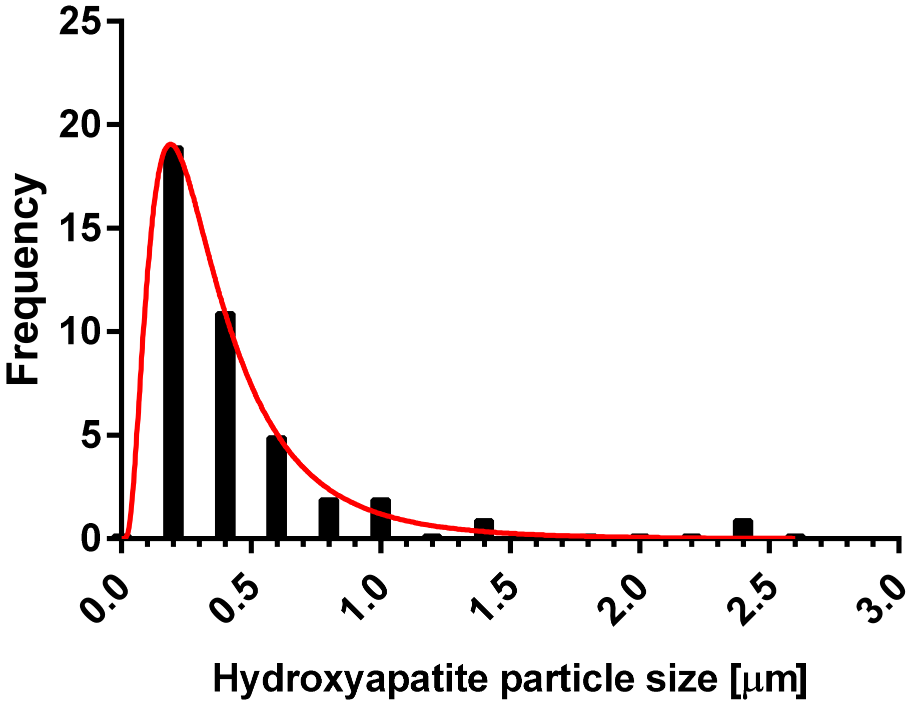

Figure S 1. Particle size distribution for HA particles.
